# Biogenic Mn-Oxides in Subseafloor Basalts

**DOI:** 10.1371/journal.pone.0128863

**Published:** 2015-06-24

**Authors:** Magnus Ivarsson, Curt Broman, Håkan Gustafsson, Nils G. Holm

**Affiliations:** 1 Department of Palaeobiology and the Nordic Center for Earth Evolution (NordCEE), Swedish Museum of Natural History, Stockholm, Sweden; 2 Department of Geological Sciences, Stockholm University, Stockholm, Sweden; 3 Department of Biomedical Engineering (MTÖ), County Council of Östergötland, Radiation Physics, Department of Medicine and Health Sciences, Linköping University, Linköping, Sweden; Fujian Agriculture and Forestry University, CHINA

## Abstract

The deep biosphere of the subseafloor basalts is recognized as a major scientific frontier in disciplines like biology, geology, and oceanography. Recently, the presence of fungi in these environments has involved a change of view regarding diversity and ecology. Here, we describe fossilized fungal communities in vugs in subseafloor basalts from a depth of 936.65 metres below seafloor at the Detroit Seamount, Pacific Ocean. These fungal communities are closely associated with botryoidal Mn oxides composed of todorokite. Analyses of the Mn oxides by Electron Paramagnetic Resonance spectroscopy (EPR) indicate a biogenic signature. We suggest, based on mineralogical, morphological and EPR data, a biological origin of the botryoidal Mn oxides. Our results show that fungi are involved in Mn cycling at great depths in the seafloor and we introduce EPR as a means to easily identify biogenic Mn oxides in these environments.

## Introduction

The subseafloor basalts have been shown to house a substantial portion of microorganisms, perhaps being the Earths largest microbial habitat [1]. Despite issues in sampling at such depths phylogenetic studies have been performed on shallow basalts [2–4] accompanied by a few studies of deeper settings [5–7] indicating the presence of both bacteria and archaea. Carbon isotope signatures of vent fluids further show that fluid circulation may support an indigenous chemosynthetic deep biosphere [8]. Additionally, drilled cores from Ocean Drilling Program (ODP) and samples from ophiolites show the presence of a highly varied fossil record ranging from ichnofossils in volcanic glass to complex fungal communities in veins and vesicles of the basalts [9–14]. Still, our knowledge of the microbial diversity, abundance and ecological role of the subseafloor biosphere is scarce.

The recognition of fungi in these environments indicates the presence of a previously neglected geobiological agent, the environmental impact of which has not yet been accounted for. In terrestrial settings, like soils for instance, fungi play a key role in mineral weathering and formation, as well as element mobilization and cycling [15]. It is probable to assume that fungi play a similar role in the subseafloor crust.

Reduced Fe, S and Mn, abundant in basalts, are considered the base for a chemolithoautotropic biosphere hosted in subseafloor basalts [16]. Fungi are heterotrophs and dependent on accessible carbohydrates for their metabolism, however, they also require essential nutrients and metals including Fe, S, and Mn for their growth [15]. While microbial oxidation of Fe(II) and formation of Fe(III) is relatively well studied [17], microbial Mn(II) oxidation and subsequent formation of Mn(III,IV) oxide minerals are less known. Formation of Mn oxides like rock varnish or marine ferromanganese nodules are often alleged to biological processes, even though the precise role of the microbes are poorly understood. Many microorganisms, especially bacteria and fungi, are known to catalyze the oxidation of Mn(II) and the formation of Mn(III,IV) oxide minerals much faster than the abiotic Mn(II) oxidation [18]. Due to this the majority of naturally occurring environmental Mn oxides are believed to be the result of biogenic Mn(II) oxidation processes [19,20]. However, distinguishing between abiotic and biotic Mn oxides is still difficult.

Electron Paramagnetic Resonance spectroscopy (EPR) is a technique to study paramagnetic species (e.g. radicals, transition metals and point defect in crystals) in an applied magnetic field [21]. Kim and coworkers [22] studied a wide range of Mn oxides including synthetic Mn oxides, natural Mn oxides with both a biological and abiotic origin, and bacteriogenic Mn oxides from controlled laboratory experiments and showed that biogenic Mn oxides has EPR signatures distinct from abiotic Mn oxides. They also showed that natural Mn oxides with a suspected biological origin had EPR signatures distinct from the abiotic Mn oxides, and thus suggested that EPR could be used to distinguish between biological and abiotic Mn oxides in natural samples.

Isolated and cultured fungi from seafloor basalts are rare but Connell and co-workers [23] reported of eight species capable of oxidizing Fe, including one fungal species capable of oxidizing Mn. A confirmation of fungal Mn oxidation at depth would significantly increase our understanding of fungi as a geobiological agent in the subseafloor crust. However, without reliable methods to sample live species at such depth identification of biogenic Mn oxides could be an approach to understand the extent of microbial Mn(II) oxidation in subseafloor basalts. Thus, means to distinguish between abiotic and biotically produced Mn oxides are required. Here, we describe Mn oxides associated with fossilized fungal communities in open vesicles of subseafloor basalts from the Detroit Seamount, Pacific Ocean. The Mn oxides have a close spatial and morphological relationship to the fungi, and to establish a purported biogenic origin we used EPR. The Mn oxides have a biogenic EPR signature suggesting a biological origin.

## Samples and Methods

The sample in the current study, 197-1204B-16R-01, 145, was collected during Ocean Drilling Program (ODP) Leg 197 at the Detroit Seamount in the Pacific Ocean. The sample was not collected in a protected area and specific permissions were not required for sampling. The field studies did not involve endangered or protected species. Detroit Seamount is of an approximate age of ~81 Ma and the sample 197-1204B-16R-01, 145 represents a depth of 936.65 metres below seafloor [24]. The sample was cut into pieces and cubes of 1X1 cm to expose vugs and fungal communities. The samples were studied in whole piece in light microscopy, Environmental Scanning Electron Microscopy (ESEM) and Raman spectroscopy for detection and identification of minerals and microfossils. Mn-oxides were further studied with Electron Paramagnetic Resonance spectroscopy (EPR) and for that small amounts of Mn-oxides were mechanically scratched out of the vugs and ground to powder. 5–10 mg was used for the analyses.

### ESEM/EDS

Environmental Scanning Electron Microscope (ESEM) and Energy Dispersive X-ray Spectroscopy (EDS) analyses were done on an FEI QUANTA FEG 650 (Oxford Instruments, UK). EDS was done using an Oxford T-Max 80 detector. The analyses were performed in low vacuum to minimize surficial charging effects. This enables the use of uncoated samples and, thus, EDS analyses of the C content. The acceleration voltage was 20 or 15 kV depending on the nature of the sample, and the instrument was calibrated with a cobalt standard. Peak and element analyses were done using INCA Suite 4.11 software and normalized to 100 wt%. Element mapping was done using Aztec software.

### Raman spectroscopy

The analyses were performed with a confocal laser Raman spectrometer (Horiba instrument LabRAM HR 800), equipped with a multichannel air-cooled (-70°C) 1024 x 256 pixel CCD (charge-coupled device) array detector. Acquisitions were obtained with an 1800 lines/mm grating. Excitation was provided by an Ar-ion laser (λ = 514 nm) source. A low laser power 1–5 mW at the sample surface was used to avoid laser induced degradation of the sample. A confocal Olympus BX41 microscope was coupled to the instrument. The laser beam was focused through a 100x objective to obtain a spot size of about 1 μm. The spectral resolution was ~0.3 cm^-1^/pixel. The accuracy of the instrument was controlled by repeated use of a silicon wafer calibration standard with a characteristic Raman line at 520.7 cm^-1^. The Raman spectra were achieved with LabSpec 5 software.

### EPR

The analysis was performed using a X-Band Bruker E500 EPR (Bruker Bio-Spin GmbH, Rheinstetten, Germany) with a 4103 TM resonator at room temperature using a clear fused quartz ERR sample tube (707-SQ-250M) from Wilmad-LabGlas (Vineland, New Jersey, USA). No EPR signal could be detected from the empty sample tube. Measurements were done using microwave power of 10 mW or 1 mW for comparisons, 2 G modulation amplitude, 5.12 ms time constant, 20 s sweep time (three added sweeps). Spectra were imported into MATLAB (version R2011a, MathWorks, Inc.) for analysis [25]. The EPR signal line width was determined as peak-to-peak, Δpp (the horizontal distance between the maximum and the minimum of a first-derivative lineshape). The EPR spectra were further analysed by comparisons with superpositions of Gaussian lines.

## Results

**Fig 1:**
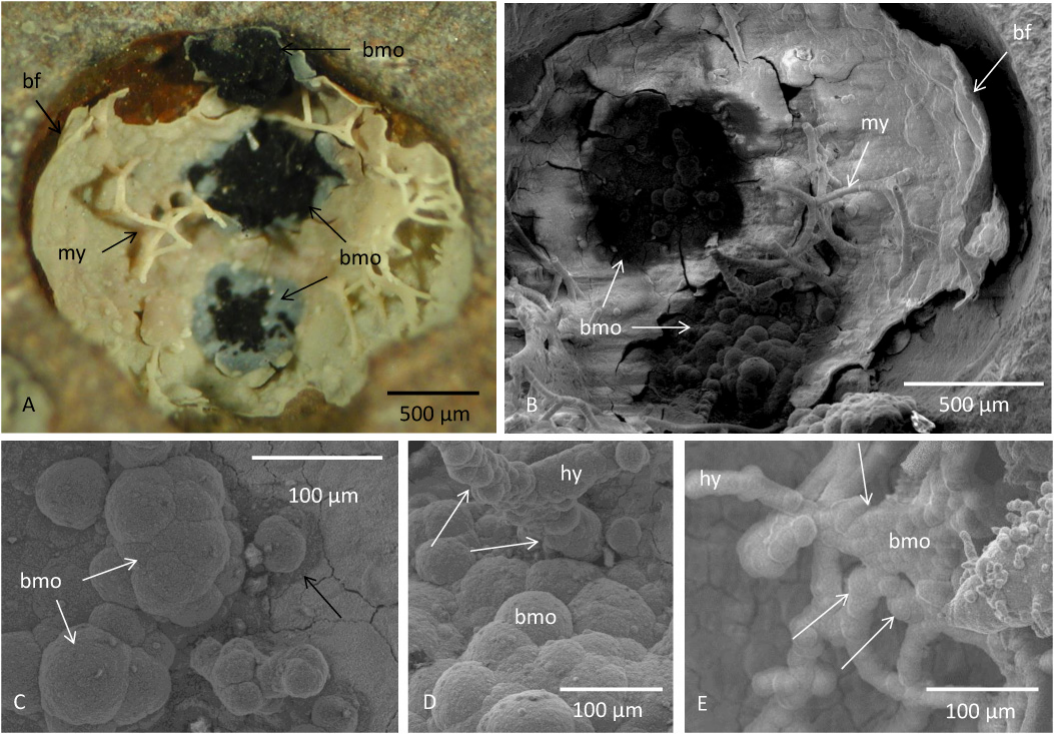
Fungal mycelium and botryoidal Mn oxides in a vug. (A) Optical microphotograph of a vug in basalt lined with a fossilized biofilm of montmorillonite from which fungal hyphae protrude to form a mycelium. Black patches are botryoidal Mn oxides. (B) ESEM image of a vug lined with fossilized biofilm from which hyphae protrude forming a mycelium. Closely associated with the mycelium are black patches of botryoidal Mn oxides. (C) ESEM image of botryoidal Mn oxides. Black arrow show the border of the Mn oxide, note the change in grayscale between the Mn oxide and the underlying montmorillonite. (D, E) ESEM images showing botryoids on the basal parts of hyphae (white arrow). Legend: bf, biofilm; my, mycelium; bmo, botryoidal Mn oxide; hy, hyphae.

**Fig 2:**
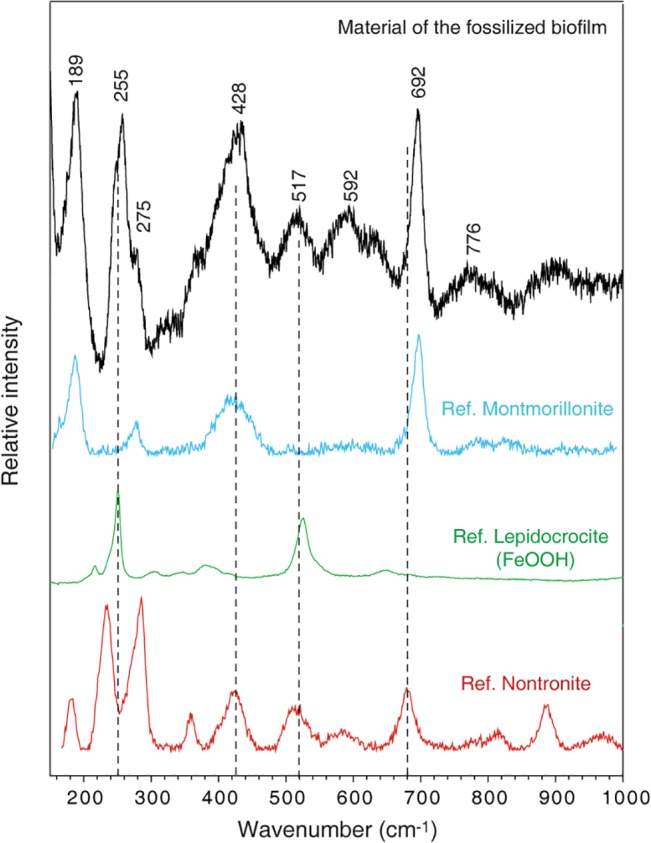
Raman spectrum of the fossilized biofilm. Raman spectrum (black) of the material that has fossilized the biofilm and hyphae is identified as Fe-rich smectite of the montmorillonite-nontronite series after comparison with RRUFF reference spectra [25]. The obtained bands are close to those of nontronite (reference spectrum in red), but exhibit small differences in peak positions. The spectrum is complex and an alternative interpretation is that the material is montmorillonite (reference spectrum in blue) and that the spectrum is influenced by the presence of small amounts of FeOOH, probably lepidocrocite (reference spectrum in green).

The investigated vugs contain fossilized fungal communities previously described and discussed according to biogenicity, fossilization and morphology [13,14]. The fungi occur as a biofilm that covers the inside of the vesicles and from which hyphae protrude and form mycelial-like networks that partly occupy the open pore space (Fig 1A and 1B). The fossilized communities including the biofilm and the hyphae are mineralized and preserved by a Fe-rich smectite of the montmorillonite-nontronite series according to RRUFF reference spectra [26]. The obtained bands are close to those of nontronite, but exhibit small differences in peak positions. The spectrum is complex and an alternative interpretation is that the material is montmorillonite that lacks Ca (Table 1) and that the spectrum is influenced by the presence of small amounts of FeOOH, probably lepidocrocite [26] (Fig 2).

**Table 1:** EDS data given in wt%.

Elements	Montmorillonite	Botryoid cross section	Botryoid cross section	Botryoid surface	Botryoid surface	Sporophore	Sporophore
O	45.38	24.43	24.25	25.27	24.52	25.12	24.14
Na	1.91	1.58					
Mg	3.06	3.93	3.40	5.40	3.27	5.80	3.11
Al	7.47	0.55	0.62	0.70	0.54	0.68	0.34
Si	28.42	0.45	0.42	0.99	0.92	0.78	0.54
Cl	0.17					0.27	
K	2.57	1.74	1.72	1.97	1.66	1.98	1.69
Ca		0.81	0.97	0.89	0.74	0.7	0.64
Ti		0.92	0.94	1.10	1.01	1.00	1.08
Mn		57.06	59.37	55.55	58.82	55.90	60.42
Fe	11.02	4.40	4.26	4.97	5.05	4.39	4.94
Co		2.79	2.68	1.24	1.61	1.67	1.83
Ni		1.34	1.36	1.91	1.84	1.70	1.26
Total	100.00	100.00	100.00	100.00	100.00	100.00	100.00

The presented measurements have been selected since they represent typical compositions of the various analyzed structures.

**Fig 3:**
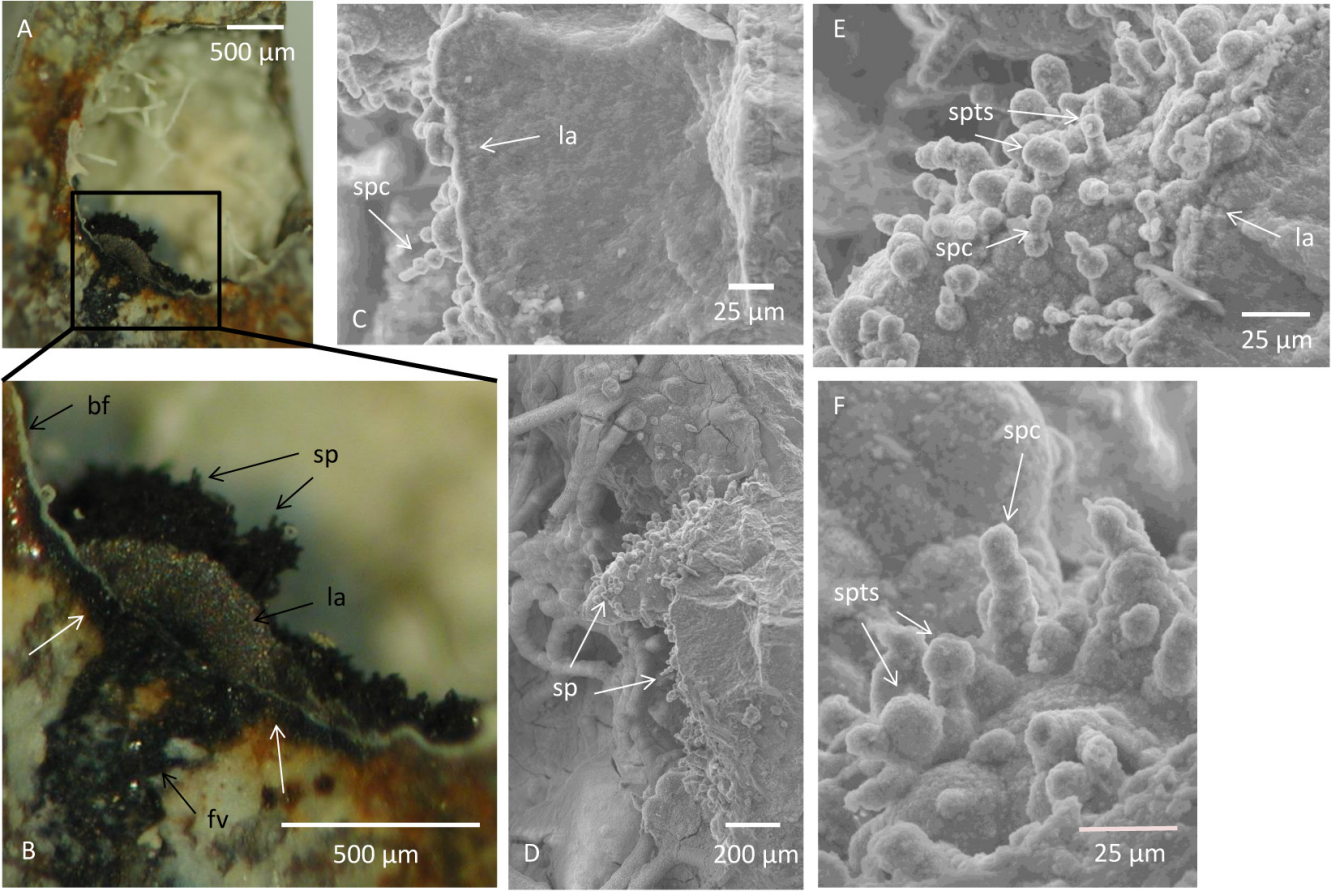
Cross sections through botryoidal Mn oxides and related sporophores. (A) Optical microphotograph of a vug with cross section through a botryoidal structure. (B) Magnified and focused part of A showing the cross section of the botryoidal Mn oxide with vague layering at the margin and sporophore-like structures on top. Black mineralized feeder veins are seen underneath the botryoidal structure. White arrows show Mn oxides formed underneath the fossilized biofilm. (C) ESEM image of a cross section through a botryoidal structure showing vague layering at the top margin and sporophores formed as separate cells stacked on each other. (D) ESEM image of a cross section through a botryoidal structure and the distribution of sporophores on its top. (E) Detailed ESEM image of D showing sporophores both made up of separate cells and terminal swelling. (F) ESEM image showing sporophores made up of separate cells and with terminal swelling. Legend: sp, sporophore; spc, sporophore with separate cells on top of each other; spts, sporophores with terminal swelling; la, layering; fv, feeder veins.

Closely associated with the fungal mycelia are botryoidal patches that consist of semi-spherical bulges varying in diameter from ~10 to ~100 μm (Fig 1). The botryoidal structures are distinguished by a black coloration that clearly defines them as dark isolated patches from the brighter montmorillonite that covers the inside of the vugs. The patches vary from ~500 μm to ~1 mm in diameter and are almost exclusively located to areas in the vugs with high abundance of hyphae. Hyphae in direct contact or closely associated with the botryoids are also often covered by botryoids at their basal parts (Fig 1B, 1D and 1E). Cross sections of the large botryoids show a vague layering at the top parts (Fig 3A–3C and 3E). Further down the layering diminish and the matrix is relative homogenous and mineralized.

Some of the surfaces of the botryoidal structures are covered by erected filamentous protrusions, 10 to 20 μm in length (Fig 3). The protrusions consist either of a few spherical structures (5–10 μm in diameter) stacked upon each other (Fig 3C, 3E and 3F) or as spherical or filamentous structures with a swelling on top (10–20 μm in diameter) (Fig 3E and 3F).

EDS analyses show that the botryoids consist of Mn-oxides with minor traces of Mg, Na, Fe, Al, Si, Ca, Ti Ni, Co, K, Cl (Table 1). Due to the working distance in ESEM the EDS analyses should be considered as indicative rather than exact. However, all analyses showed similar chemical composition, thus, the measured elements are correct but the values should be treated more as an indication than precise. EDS analyses also showed that both the surfaces and the cross sections of the botryoids, as well as the protrusions on top of the botryoids are similar in composition with only minor variations among the trace elements.

In a few cases where the botryoids have been successively split it is possible to see that they are fed from beneath by veins partly filled with Mn-oxides (Fig 3A and 3B). If this is the case for all botryoids is not possible to determine but highly likely. The Mn was probably introduced through micro-cracks to the vesicles as soluble Mn(II) in the fluids. At places of the feeder veins Mn oxides have formed above the biofilm but also intruded underneath which indicate that the biofilm was laid down prior to the introduction of Mn(II) and the subsequent oxidation to form Mn (III,IV) oxide minerals.

**Fig 4:**
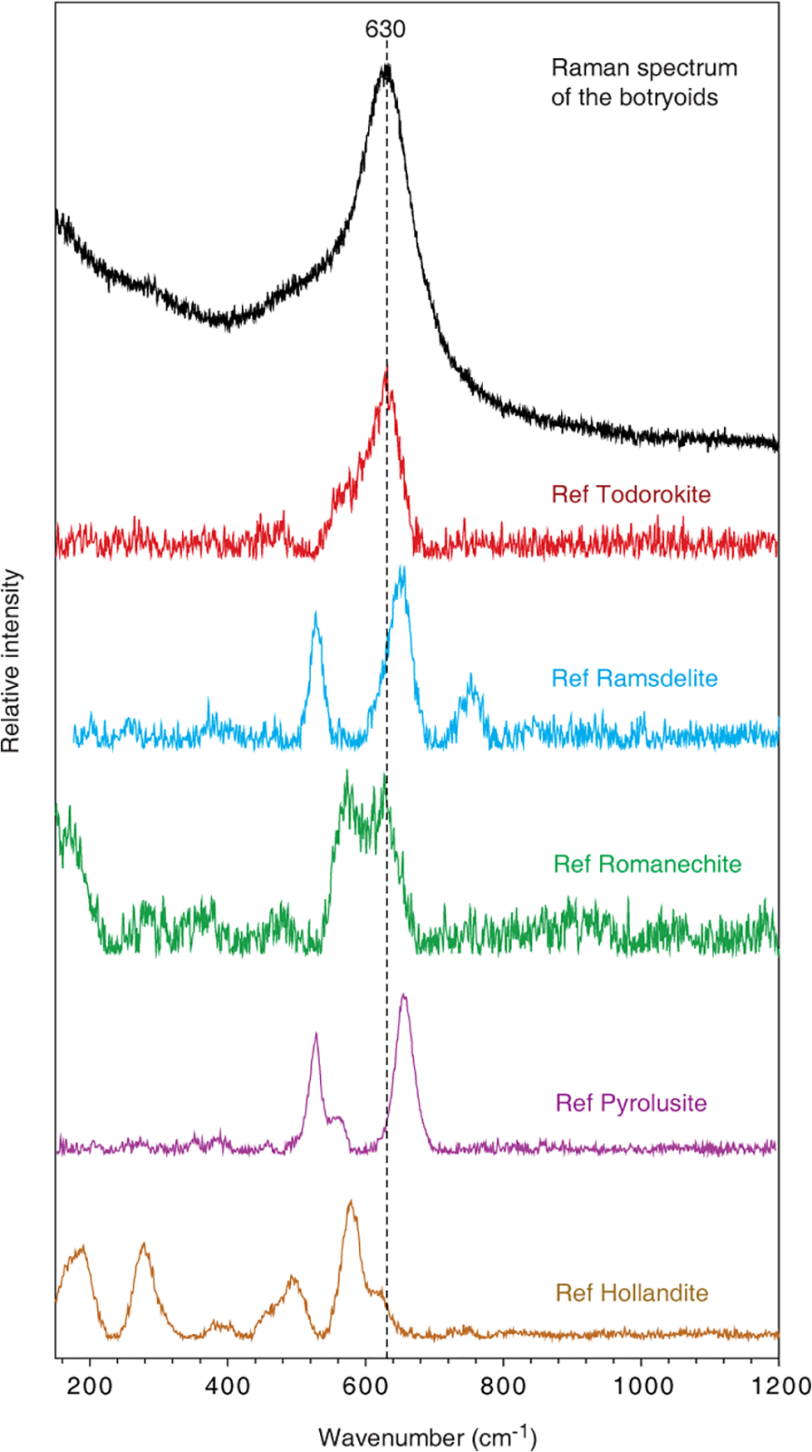
Raman spectrum of the botryoids. Raman spectrum (black) of the botryoidal structure identified as todorokite after comparison with RRUFF reference spectra of manganese dioxides from Downs (2006).

Raman measurements were done on the surfaces of the botryoids, on the protrusions and at cross sections with the same result. The obtained Raman spectrum of the botryoids is within the range of Mn-oxides and, thus, confirms the EDS measurements. The spectrum displays a band at 630 cm^-1^ (Fig 4). Manganese dioxides typically have characteristic bands in the range 480–700 cm^-1^ (RRUFF database [26]) and a comparison of 344 different spectra from Mn-bearing phases, of which five are illustrated in Fig 4, shows that the obtained spectrum is similar to the spectrum of todorokite. The general chemical formula for todorokite is (Na, Ca, K, Ba, Sr)_1-x_(Mn, Mg, Al)_6_O_12_·3-4H_2_O, which corresponds relatively well with the EDS analyses except for the Ba and Sr that was not detected.

**Fig 5:**
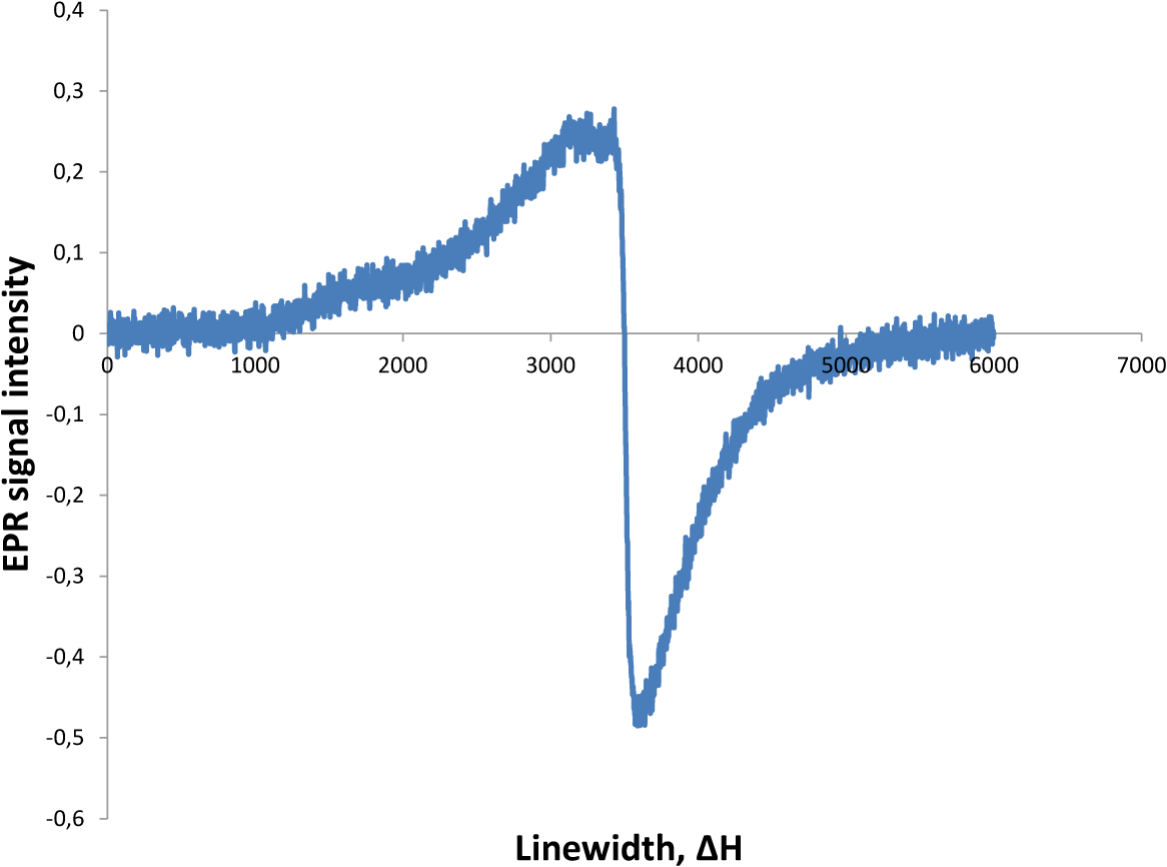
EPR spectra of the todorokite.

**Fig 6:**
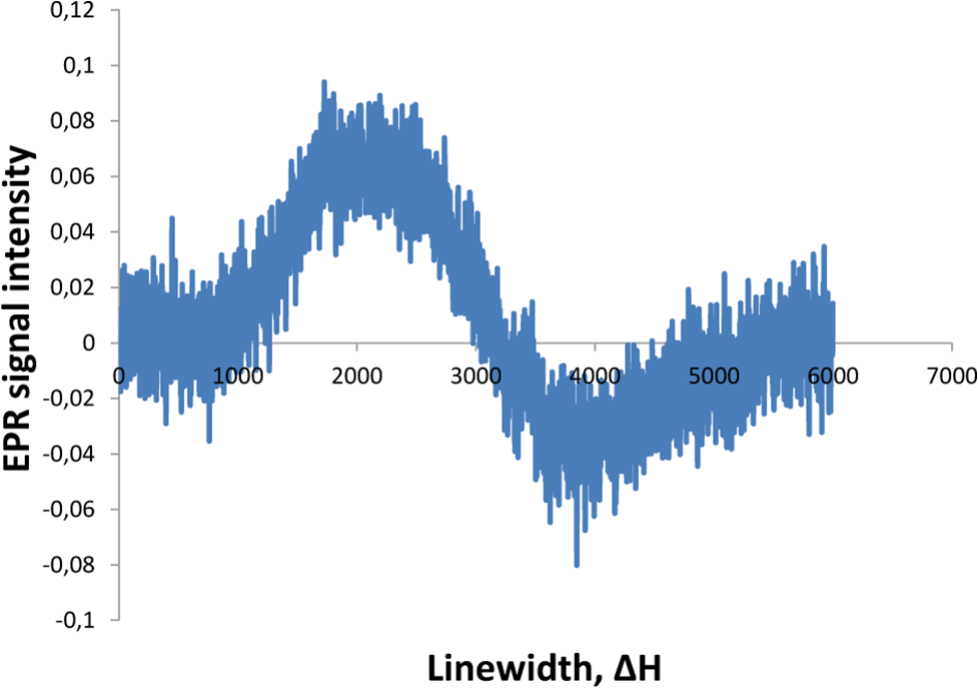
EPR spectra for abiotic Mn oxide.

EPR measurements of the Mn-oxide gave an anisotropic signal centered at g = 1.99, and a line width (peak-to-peak) of approximately Δpp = 500 G (Fig 5). As an abiotic reference we used a sample from an epithermal vein from the Vani hydrothermal system, Milos Island Greece. This vein is part of a feeder vein system for a Mn ore and contains Mn minerals of purported abiotic origin. EPR measurements gave a signal centered at g = 2.29, and a line width (peak-to-peak) of approximately Δpp = 1900 G (Fig 6).

## Discussion

### The botryoids

The biogenicity of the fungal hyphae and mycelium has been discussed and established in previous reports [13,14], and we will therefore not discuss those structures further. The additional structures described in the current report have a close spatial and morphological relationship to the fungal communities, which is especially pronounced where smaller spherical botryoids cover the basal parts of hyphae, and where the upright protrusions on the botryoidal surfaces occur. The protrusions are characterized by erected projections of similar height across a surface and are segmented in individual cell-like structures, sometimes with a terminal swelling. In a fungal context, such club-shaped protrusions correspond to reproduction structures like sporophores [14]. Conidia, for example, asexual spores among Ascomycetes, are characterized by septated hypha that form by budding of undifferentiated hyphae, and in some species the terminal cell enlarges to form a conidium [27,28]. Fungal sporophores and conidia are diverse in form and structure but the general features are characteristic and represent the best explanation for the current protrusions.

The close spatial relationship to both hyphae and sporophores indicate that the botryoids are an integral constituent of the fungal community. They are probably not an organismal structure as fruiting bodies or resting structures but more likely a mineralization formed by the presence of the microorganisms. The precipitation of the Mn oxides on the basal parts of the hyphae shows that the hyphal mycelium predated the formation of Mn oxides. However, the presence of sporophore-like structures on top of the Mn oxides indicates that the fungal community still was active after the formation of the Mn oxides. It is thus most likely that the fungi existed more or less contemporaneous with the formation of the Mn oxides in the vugs.

Todorokite is, together with buserite and birnessite, some of the most commonly preserved Mn oxides and frequently invoked as a common biogenic mineral formed from microbial Mn(II) oxidation [29]. It is also a common constituent of ferromanganese nodules [30]. Even though there are distinct differences in occurrence and morphology between the botryoids and deep-sea ferromanganese nodules the environment is somewhat similar and a biological involvement is alleged for both [29].

Terrestrial basalts can sometimes be covered by rock varnish, so called varnished basalt [31]. The Raman spectra of varnished surface coatings on the basalt are characterized by a main band in the ~620–630 cm^-1^ wavenumber range with a broad base [31] which corresponds very well to the spectra of our botryoidal structures. The comparison to rock varnish is perhaps not valid since it is a product of terrestrial processes and often found in dry desert environments. However, rock varnish, in general, is a thin, sometimes layered crust of mainly Mn-oxides with an alleged biological origin [32–34]. Especially, micro-colonial fungi seems to play an important role in its formation, thus, there is a striking resemblance in localized fungal colonization on a rock substrate and formation of an associated Mn oxide crust, and a comparison is applicable even though the environments differ.

### Biogenic formation of Mn oxides in subseafloor basalt

Abiotic oxidation of Mn(II) is slow up to pH 8.5 and requires years for completion [29]. Many microorganisms, especially bacteria and fungi, are known to catalyze the oxidation of Mn(II) and the formation of Mn(III,IV) oxide minerals much faster than the abiotic Mn(II) oxidation. Besides, a number of field studies have shown that biological processes are responsible for Mn(II) oxidation [19]. Due to these reasons, the majority of naturally occurring environmental Mn oxides are believed to be the result of biogenic Mn(II) oxidation processes [18–20,29,35]. However, distinguishing between abiotic and biotic Mn oxides is still difficult.

Biogenic Mn oxides produce electron paramagnetic resonance (EPR) spectral signatures that are distinct from abiogenic Mn oxides [22]. Abiogenic Mn oxides have linewidths ΔH>1200 G, suspected biominerals like desert varnish and Mn nodules have linewidths 600 G < ΔH < 1200 G, and biogenic Mn oxides ΔH <560 G [22]. Also, abiogenic Mn oxides have widely scattered *g*-values while biogenic Mn oxides cluster around *g* = 2.0. Thus, our EPR measurement with *g* at 1.99, and a line width of 500 G correlate very well to biogenic Mn oxides. Also, our abiotic sample with a line width of 1900 G, and *g* at 2.29 correspond very well to abiotic Mn oxides and confirm the validity of EPR as a method to distinguish between abiotic and biogenic Mn oxides.

The difference in linewidths between biogenic Mn oxides with narrow linewidths at ΔH <560 G and abiotic Mn oxides that range from 1200 to 4000 G in linewidths is due to structural characteristics [22]. Mn oxides with few cation vacancies and mixed ionic states (Mn(III) and Mn(IV)) will show broader linewidths through larger dipolar interactions. Abiotic Mn oxides have a relatively high Mn(III) content as well as significant structural Mn(II) content and a low vacancy content. Biogenic Mn oxides, on the other hand, with more site vacancies and little Mn(III) will have smaller dipolar interactions and moderate exchange narrowing, the combination leading to narrower linewidths. In natural samples, the vacancies of biogenic Mn oxides will gradually fill with various cations. The current todorokite, for instance, contains minor Fe, Ni, Co that could be due to subsequent filling by cations. However, the narrow EPR linewidths do not seem to have substantially been affected by this. Desert varnish and Mn nodules also maintain a narrow linewidths despite later addition of cations, Fe oxides, or even transformation from a layered mineral structure to a tunnel structure [22].Microbially produced Mn oxides are usually relative amorphous δ-MnO_2_-like precursors, which suggest that the current todorokite has been diagenetically mineralized. However, aging and subsequent transformation of Mn oxides occur with relative minor modification to the original EPR biosignature [22,29].

When adding the EPR data to the morphological and mineralogical data a biogenic origin of the todorokite seems most likely, however, it is difficult to distinguish if the Mn oxide is formed by the fungi or a symbiotic prokaryote not observed, and perhaps not even preserved. Both bacteria and fungi are known to catalyze Mn(II) oxidation and form Mn(III,IV) oxides [18,19,29]. In deep-sea settings Mn(II) oxidizing bacteria are diverse and include species from both α- and γ-Proteobacteria [29,36]. Fungi is much less explored in subseafloor environments but have in the last decade been found in deep-sea sediments [37,38], at hydrothermal vents [20,39], and fossilized in the subseafloor crust [9,12–14]. Fungi have only been found, isolated and successfully cultured once from seafloor basalt rock surface [23]. The fungi were isolated from active Fe-oxide mats and most isolates were found to produce siderophores. One species, *Rhodotorula graminis*, oxidized Mn(II) to Mn(IV) oxide minerals [23]. *Rhodotorula graminis* is a Basidiomycete able to form true hyphae and mycelial mats *in vitro* [40], and was shown to be a strong siderophore producer [23]. If the current fungal community is related to *Rhodotorula graminis* or not is difficult to assess from fossilized material but it is evident that the fungal communities have been involved in the formation of Mn oxide minerals and that the Mn oxidation processes has been extensive in this particular sample. Microbial Mn oxidation require dissolved oxygen, which potentially could be a limiting factor in these environments. In general, the distribution of dissolved oxygen in subseafloor basalts is poorly understood due to technical limitations in sampling and monitoring. Dissolved oxygen is introduced to the oceanic crust by seawater recharge at basaltic outcrops, and its further propagation through the system is controlled by the present fluid regime. Thus, the extension and longevity of dissolved oxygen is controlled by a range of various parameters like sediment cover, permeability, porosity and depth and age of the crust. Besides, oxygen has the highest redox potential of all electron acceptors and is readily consumed by fluid-rock interactions and microbial activity [41]. The oxygen consumption rate in marine sediments, for instance, is reflected by the overall microbial activity, thus, in sediments with moderate to high content of organic matter oxygen is consumed in the first few millimetres to centimeters, whereas in organic-poor sediments oxygen can persist for meters [42]. Oxygen consumption in subseafloor basalts is poorly constrained, however circulation of oxic fluids occur through cool regions [41]. At North Pond, the western flank of the Mid-Atlantic Ridge, deep anoxic sediments are oxygenized due to upflow of oxic fluids from the underlying igneous crust. Orcutt et al. [43] further calculated oxygen consumption rates of 1nmol cm^-3^_ROCK_ d^-1^ or less in the upper sections of the young (~8Ma), and cool (<25°C) basaltic crust at North Pond. Just like ridge-flanks, seamounts are areas where the igneous crust is exposed, and where much of the fluid exchange between the ocean and the basement is focused [44,45]. Compared to areas of the ocean floor covered by sediments there is a continuous recharge of oxic fluids at seamounts of which the Mn oxides at Detroit Seamount bear witness of.

The mineral succession and occurrence of fossilized fungi in the samples from Detroit Seamount indicates that the existence of an active fungal community and the precipitation of Mn oxides were contemporaneous. The Mn was introduced by micro-cracks to the vugs as soluble Mn(II) where it was readily oxidized to Mn(III,IV) oxide minerals due to the microbial presence. The fungi might have been solely responsible for the formation of Mn oxide minerals. Extensive production of siderophores could have scavenged Mn(II) from the fluids and mediated oxidation to Mn(III,IV) oxide minerals. However, fungi have been shown to exist in spatial and symbiotic-like relationships with prokaryotes in subseafloor basalts [14], and even though no clear remains are observed of symbiotic prokaryotes we can not exclude the possibility of a symbiosis with a Mn oxidizing prokaryote responsible for some or all Mn oxidation. As the case with many other Mn oxides, like ferromanganese nodules or rock varnish, microorganisms seems to have been actively involved in the formation of the botryoidal todorokite but the exact role of the microbes is unclear.

## Conclusions

Botryoidal Mn oxides consisting of todorokite observed in vugs of subseafloor basalt from the Detroit Seamount, Pacific Ocean, are suggested to be biological in origin. This interpretation is based on mineralogical, morphological and EPR data. There is a close relationship both spatially and in terms of time indicating that the fungi were contemporaneous with the formation of the Mn oxides.

Our results show that microbial, possibly fungal, mediated oxidation of Mn(II) and subsequent formation of Mn(IV) oxide minerals not only are restricted to the seafloor and hydrothermal vents but also occur at depth in subseafloor basalts. This has implications for geobiological cycling of Mn in these systems and especially emphasizes the role of fungi as a geobiological agent and promoter of biomineralization. We further show the advantage of using EPR in identification of biogenic Mn oxides.

## References

[1] Schrenk MO, Huber JA, Edwards KJ. Microbial provinces in the subseafloor. A. Rev. Marine Sci. 2009; 2: 279–304. 10.1146/annurev-marine-120308-081000 21141666

[2] Thorseth IH, Torsvik T, Torsvik V, Daae FL, Pedersen RB. Keldysh-98 Scientific Party. Diversity of life in ocean floor basalt. Earth Planet. Sci. Lett. 2001; 194: 31–37.

[3] Lysnes K, Thorseth IH, Steinsbau BO, Øvreås L, Torsvik T, Pedersen RB. Microbial community diversity in seafloor basalt from the Arctic spreading ridges. FEMS Microbiol. Ecol. 2004; 50: 213–230. 10.1016/j.femsec.2004.06.014 19712362

[4] Santelli CM, Orcutt BN, Banning E, Bach W, Moyer CL, Sogin ML, et al Abundance and diversity of microbial life in ocean crust. Nature 2008; 453: 653–657. 10.1038/nature06899 18509444

[5] Orcutt BN, Bach W, Becker K, Fisher AT, Hentscher M, Toner BM, et al Colonization of subsurface microbial observatories deployed in young ocean crust. ISME J. 2010; 5: 692–703. 10.1038/ismej.2010.157 21107442 PMC3217339

[6] Mason OU, Nakagawa T, Rosner M, Van Nostrand JD, Zhou J, Maruyama A, et al First investigation of microbiology of the deepest layer of ocean crust. PLoSONE 2010; 5: e15399 10.1371/journal.pone.0015399 PMC2974637 21079766

[7] Lever MA, Rouxel A, Alt JC, Shimizu N, Ono S, Coggon RM, et al Evidence for microbial carbon and sulphur cycling in deeply buried ridge flank basalt. Science 2013; 339: 1305–1308. 10.1126/science.1229240 23493710

[8] McCarthy MD, Beaupré SR, Walker BD, Voparil I, Guilderson TP, Druffel ERM. Chemosynthetic origin of ^14^C-depleted dissolved organic matter in a ridge-flank hydrothermal system. Nat. Geosci. 2011; 4: 32–36.

[9] Schumann G, Manz W, Reitner J, Lustrino M. Ancient fungal life in North Pacific eocene oceanic crust. Geomicrobiol. J. 2004; 21: 241–246.

[10] Staudigel H, Furnes H, McLoughlin N, Banerjee NR, Connell LB, Templeton A. 3.5 billion years of glass bioalteration: Volcanic rocks as a basis for microbial life? Earth-Sci. Rev. 2008; 89: 156–176

[11] Peckmann J, Bach W, Behrens K, Reitner J. Putative cryptoendolithic life in Devonian pillow basalt, Rheinisches Schiefergebirge, Germany. Geobiology 2008; 6: 125–135. 10.1111/j.1472-4669.2007.00131.x 18380875

[12] Ivarsson M, Bengtson S, Belivanova V, Stampanoni M, Marone F, Tehler A. Fossilized fungi in subseafloor Eocene basalts. Geology 2012; 40: 163–166.

[13] Ivarsson M, Bengtson S, Skogby H, Belivanova V, Marone F. Fungal colonies in open fractures of subseafloor basalt. Geo-Mar. Lett. 2013; 33: 233–243.

[14] Bengtson S, Ivarsson M, Astolfo A, Belivanova V, Broman C, Marone F, et al Deep-biosphere consortium of fungi and prokaryotes in Eocene sub-seafloor basalts. Geobiology 2014; 12: 489–496. 10.1111/gbi.12100 25214186

[15] Gadd GM. Geomycology: biogeochemical transformations of rocks, minerals, metals and radionuclides by fungi, bioweathering and bioremediation. Myc. Res. 2007; 111: 3–49. 10.1016/j.mycres.2006.12.001 17307120

[16] McCollom TM. Geochemical constraints on sources of metabolic energy for chemolithoautotrophy in ultramafic-hosted deep-sea hydrothermal systems. Astrobiology 2007; 7: 933–950. 10.1089/ast.2006.0119 18163871

[17] Ehrlich HL. Geomicrobiology. New York: Marcel Dekker 2002.

[18] Tebo BM, Ghiorse WC, van Waasbergen LG, Siering PL, Caspi R. Bacterially mediated mineral formation: insighths into managanese(II) oxidation from molecular genetic and biochemical studies. Geomicrobiology: interactions between microbes and minerals, Revs. Min. 1997; 35225–266.

[19] Tebo BM, Bargar JR, Clement BG, Dick GJ, Murray KJ, Parker D, et al Biogenic manganese oxides: properties and mechanisms of formation. Annu. Rev. Earth Planet. Sci. 2004; 32: 287–328.

[20] Bargar JR, Fuller CC, Marcus MA, Brearley AJ, De la Rosa MP, Webb SM, et al Structural characterization of terrestrial microbial Mn oxides from Pinal Creek, AZ. Geochim. Cosmochim. Acta. 2009; 73: 889–910.

[21] Weil JA, Bolton JR. Electron paramagnetic resonance: elementary theory and practical applications Wiley, Hoboken, 2007.

[22] Kim SS, Bargar JR, Nealson KH, Flood BE, Kirschvink JL, Raub TD, et al Searching for biosignatures using electron paramagnetic resonance (EPR) analysis of manganese oxides. Astrobiology 2011; 11: 775–786. 10.1089/ast.2011.0619 21970705

[23] Connell L, Barrett A, Templeton A, Staudigel H. Fungal diversity associated with an active deep sea volcano: Vailulu’u Seamount, Samoa. Geomicrobiol. J. 2009; 26: 597–605.

[24] Tarduno JA, Duncan RA, Scholl DW. Leg 197 summary. Proc. ODP, Init. Rep. 2002; 197: 1–92.

[25] Stoll S, Schweiger A. EasySpin, a comprehensive software package for spectral simulation and analysis in EPR. J. Magn. Reson. 2006; 178: 42–55. 16188474 10.1016/j.jmr.2005.08.013

[26] Downs RT. The RRUFF Project: an integrated study of the chemistry, crystallography, Raman and infrared spectroscopy of minerals. Program and Abstracts of the 19th General Meeting of the International Mineralogical Association in Kobe, Japan. (2006; O03–13.

[27] Webster J, Weber RWS. Introduction to fungi Third edition Cambridge University Press, Cambridge, UK 2007.

[28] Ivarsson M. The subseafloor basalts as fungal habitats. Biogeosciences; 2012: 9, 3625–3635.

[29] Tebo BM, Johnson HA, McCarthy JK, Templeton AS. Geomicrobiology of manganese(II) oxidation. TRENDS Microbiol. 2005; 13: 421–428. 16054815 10.1016/j.tim.2005.07.009

[30] Burns RG, Mee Burns V. Manganese oxides In Burns R.G. (ed.) Marine Minerals, Mineralogical Society of America Short Course Notes Vol. 6, Mineralogical Society of America, Washington, D.C. 1979; 1–46.

[31] Israel EJ, Arvidson RE, Wang A, Pasteris JD, Joliff BL. Laser Raman spectroscopy of varnished basalt and implications for in situ measurements of Martian rocks. J. Geophys. Res. 1997; 102 (E12): 28705–28716.

[32] Gorbushina AA, Krumbein WE, Volkmann M. Rock surfaces as life indicators: new ways to demonstrate life and traces of former life. Astrobiology 2002; 2: 203–213. 12469369 10.1089/15311070260192273

[33] Staley JT, Palmer F, Adams JB. Microcolonial fungi: common inhabitants on desert rocks? Science 1982; 215: 1093–1095. 17771840 10.1126/science.215.4536.1093

[34] Taylor-George S, Palmer FE, Staley JT, Borns DJ, Curtiss B, Adams JB. Fungi and bacteria involved in desert varnish formation. Microb. Ecol. 1983; 9, 227–245. 10.1007/BF02097739 24221703

[35] Miyata N, Tani Y, Sakata M, Iwahori K. Review: microbial manganese oxide formation and interaction with toxic metals. J. Biosci. Bioeng. 2007; 104: 1–8. 17697976 10.1263/jbb.104.1

[36] Templeton AS, Staudigel H, Tebo BM. Diverse Mn(II)-oxidizing bacteria isolated from submarine basalts at Loihi Seamount. Geomicrobiol. J. 2005; 22: 127–139.

[37] Orsi W, Biddle JF, Edgcomb V. Deep sequencing of subseafloor eukaryotic rRNA reveals active fungi across marine subsurface provinces. PLoS ONE 2013; 8: 1–10. 10.1371/journal.pone.0056335 PMC3572030 23418556

[38] Orsi WD, Edgcomb VP, Christman GD, Biddle JF. Gene expression in the deep biosphere. Nature 2013; 499: 205–208. 10.1038/nature12230 23760485

[39] López-Garcia P, Vereshchaka A, Moreira D. Eukaryotic diversity associated with carbonates and fluid-seawater interface in Lost-City hydrothermal field. Environ. Microbiol. 2007; 9: 546–554. 17222152 10.1111/j.1462-2920.2006.01158.x

[40] Kurtzman C, Fell JW. The yeasts: a taxonomic study Elsevier, New York 1998.

[41] Bach W, Edwards KJ. Iron and sulphide oxidation within the basaltic ocean crust: Implications for chemolithoautotrophic microbial biomass production. Geochim. Cosmochim. Acta 2003; 67: 3871–3887.

[42] Orcutt BN, Sylvan JB, Knab NJ, Edwards KJ. Microbial ecology of the dark ocean above, at, and below the seafloor. Microbiol. Mol. Biol. Rev. 2011; 75: 361–422. 10.1128/MMBR.00039-10 21646433 PMC3122624

[43] Orcutt BN, Wheat CG, Rouxel O, Hulme S, Edwards KJ, Bach W. Oxygen consumption rates in subseafloor basaltic crust derived from a reaction transport model. Nat. Comm. 2013; 4:2539, 10.1038/ncomms3539 24071791

[44] Fisher AT, Wheat CG. Seamounts as conduits for massive fluid, heat, and solute fluxes on ridge flanks. Oceanography 2010; 23: 74–87.

[45] Bekins BA, Spivack AJ, Davis EE, Mayer LA. Dissolution of biogenic ooze over basement edifices in the equatorial Pacific with implications for hydrothermal ventilation of the oceanic crust. Geology 2007, 35:679–682.

